# Observation of ***ν*** = 5/2 Fractional Quantum Hall Effect in Trilayer Graphene Proximitized by V‐Doped WSe_2_

**DOI:** 10.1002/adma.202514268

**Published:** 2025-09-13

**Authors:** Pramod Ghising, Ashok Mondal, Mallesh Baithi, Jongchan Kim, Jieun Lee, Kenji Watanabe, Takashi Taniguchi, Young Hee Lee

**Affiliations:** ^1^ Center for Integrated Nanostructure Physics Sungkyunkwan University Suwon 16419 Republic of Korea; ^2^ Center for Low‐Dimensional Quantum Materials Hubei University of Technology Wuhan 430062 China; ^3^ Center for Two‐dimensional Quantum Heterostructures Institute for Basic Science (IBS), Sungkyunkwan University Suwon 16419 Republic of Korea; ^4^ Department of Energy Science Sungkyunkwan University Suwon 16419 Republic of Korea; ^5^ Department of Physics and Astronomy and Institute of Applied Physics Seoul National University Seoul 08826 Republic of Korea; ^6^ Research Center for Electronic and Optical Materials National Institute for Materials Science 1‐1 Namiki Tsukuba 305‐0044 Japan; ^7^ Research Center for Materials Nanoarchitectonics National Institute for Materials Science 1‐1 Namiki Tsukuba 305‐0044 Japan

**Keywords:** dirac gullies, fractional quantum hall effect, magnetic proximity effect, non‐Abelian anyons, reentrant integer quantum Hall effect, strongly correlated physics, trilayer graphene, V‐doped WSe_2_

## Abstract

Graphene has long been a test bed for observing strongly correlated phenomena owing to its 2D nature and ability to host high carrier mobility. However, the lack of intrinsic magnetism and weak spin‐orbit coupling limits its ability to host strong electronic correlations. Here, observation of strongly correlated phenomena in trilayer graphene (TLG) proximitized by a ferromagnetic V‐doped WSe_2_ (V‐WSe_2_) overlayer is reported. These include the emergence of an odd‐ and even‐denominator fractional quantum Hall state at ***ν***  = 5/2 and reentrant integer quantum Hall effect in the hole regime of the TLG, driven by proximate magnetism from the V‐WSe_2_. A remarkably large activation energy gap (**Δ**_5/2_ = 48 ± 5 K) for the 5/2 fractional state is observed, which is essential for probing its non‐Abelian nature. Furthermore, the large **Δ**_5/2_ significantly enhances its feasibility for topological quantum computation by exponentially suppressing the error rates. Additionally, the formation of three additional Dirac cones, termed Dirac “gullies,” is observed, which manifest as threefold‐degenerate Landau levels in magnetotransport measurements. These findings not only advance the role of magnetism in graphene‐based heterostructures but also open pathways toward studying non‐Abelian quasiparticles for their exotic fundamental and technological implications.

## Graphical abstract

Even‐denominator 5/2 fractional state in trilayer graphene/V‐doped WSe_2_ heterostructure, mediated by proximitized magnetism, is demonstrated. An exceptionally high energy gap ∆_5/2_ (48 K) for the 5/2 state, significantly surpassing previous values in semiconductors and bilayer graphene (∆_5/2_ < 1 K) is reported. The large ∆_5/2_ is essential for probing the non‐Abelian nature of the 5/2 state and has implications for fault‐tolerant topological quantum computation.

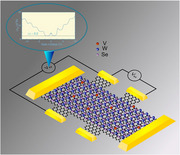

## 1 Introduction

The discovery of the integer quantum Hall effect (IQHE) in 2D electron gas (2DEG) in semiconductors^[^ ^1^ ^]^ has extended the realm of quantum phenomena.^[^ ^2^ ^]^ Under a strong perpendicular magnetic field, the 2DEG system exhibits quantized Landau levels (LLs) with the Hall conductivity quantized in integer multiples of $\frac{e^{2}}{h}$ (where *e* is the electron charge and *h* is the Planck constant). Beyond these integer quantization effects, electron interactions within the LLs give rise to a plethora of strongly correlated phenomena, including fractional quantum Hall effect (FQHE),^[^ ^3^, ^4^ ^]^ Wigner crystals,^[^ ^5^, ^6^, ^7^ ^]^ and bubble and stripe charge density waves.^[^ ^8^, ^9^ ^]^ Competition among these correlated phases can further result in emergent phenomena such as the reentrant integer quantum Hall effect (RIQHE).^[^ ^10^, ^11^ ^]^

FQHE features partially filled LLs, where strong electron interaction prompts quantized Hall plateaus to appear at fractional filling factor ν  =  *p*/*q* (*p* and *q* are both integers). Theoretical description of the fractional states with odd denominator is provided by Laughlin's wave function^[^ ^4^ ^]^ and Jain's composite fermion model.^[^ ^12^ ^]^ Meanwhile, the even denominator state at ν  = 5/2 is considered to be exotic.^[^ ^13^, ^14^ ^]^ The Moore‐Read theory^[^ ^15^ ^]^ describes the 5/2 state as a type of p‐wave superconductor, arising from Cooper pairing of composite fermions.^[^ ^16^, ^17^, ^18^ ^]^ Moreover, the nature of quasiparticles in the 5/2 state is postulated to be non‐Abelian anyons and a host for Majorana modes,^[^ ^19^, ^20^ ^]^ which have garnered significant interest for their potential applications in fault‐tolerant topological quantum computation.^[^ ^21^, ^22^ ^]^

The 5/2 fractional state was first observed in GaAs 2DEG systems.^[^ ^13^ ^]^ However, experimental demonstrations of its exotic properties remain challenging due to its fragile nature.^[^ ^16^, ^23^ ^]^ Theoretically, the 5/2 state is predicted to exhibit greater stability in bilayer graphene compared to GaAs 2DEG systems.^[^ ^24^ ^]^ Indeed, the 5/2 state has been experimentally observed in bilayer graphene.^[^ ^16^, ^17^ ^]^ The observation of FQHE in graphene^[^ ^16^, ^25^, ^26^, ^27^, ^28^ ^]^ have fueled the search for 5/2 fractional state in monolayer and trilayer graphene as well. However, the 5/2 state has remained elusive in monolayer and trilayer graphene until now.

The activation energy gap Δ of fractional states is a measure of their robustness.^[^ ^29^ ^]^ Higher Δ values are particularly valuable for experimentally probing the non‐Abelian statistics of quasiparticles (by braiding the quasiparticles) arising in even‐denominator fractional states.^[^ ^30^ ^]^ In high‐quality GaAs 2DEG systems, the measured Δ_5/2_​ is very small,^[^ ^19^, ^31^, ^32^ ^]^ while the highest reported value of Δ_5/2_ is only close to ≈1 K in bilayer graphene.^[^ ^16^ ^]^ The small Δ_5/2_ remains a major setback for experimentally exploring non‐Abelian physics of the 5/2 state. In this study, we fabricated ABA‐trilayer graphene (TLG) proximitized by a ferromagnetic 0.1% V‐doped WSe_2_ (V‐WSe_2_). The TLG/V‐WSe_2_ heterostructure is encapsulated within hexagonal boron nitride (h‐BN) layers. We report the observation of strongly correlated phases of RIQHE and FQHE in TLG/V‐WSe_2_ at 2 K. Notably, we observe a remarkably high value of Δ_5/2_ = 48 ± 5 K for the even‐denominator ν  =  5/2 state in the hole regime under perpendicular magnetic fields of 7–8 T, despite a relatively low carrier mobility (∼7 × 10^3^ cm^2^ V^−1^ s^−1^). Additionally, our samples exhibit a strong trigonal warping‐induced anomalous LL filling sequence.

## 2 Results and Discussion

**Figure 1:**
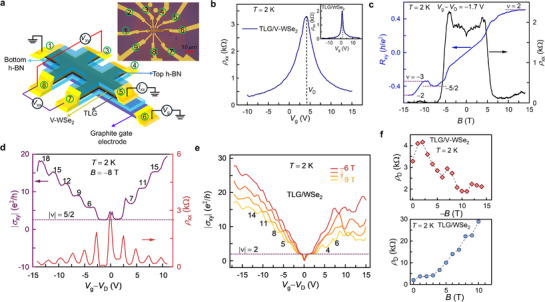
FQHE in TLG/V‐WSe_2_. a) Device schematic of the TLG/V‐WSe_2_ heterostructure. The inset shows the device image; the scale bar is 10 µm. The different electrodes are identified by the encircled numbers. b) Longitudinal resistivity ρ_xx_ as a function of *V*_g_ at *B* = 0 T and *T* = 2 K. Shift of the ρ_xx_ peak to positive voltage implies a p‐doped TLG channel. The inset shows ρ_xx_ of a pristine TLG/WSe_2_ control sample at *B =* 0 T. c) *R*_xy_ (left axis) and ρ_xx_ (right axis) as a function of magnetic field at *V*_g_− *V*_D_ = − 1.7 V and *T* = 2 K. IQHE states, along with strongly correlated phases of FQHE and RIQHE, are clearly visible as plateaus (denoted by dashed lines). d) |σ_xy_| and ρ_xx_ as a function of *V*_g_ at *B*  =   − 8 T and *T* = 2 K. The 5/2 fractional and the integer states are marked by distinct plateaus in |σ_xy_| with a corresponding minimum in ρ_xx_. Integer plateaus on the hole side appear in steps of $\frac{3e^{2}}{h}$. This threefold degeneracy is evidence of Dirac gullies in the TLG/V‐WSe_2_ sample. e) |σ_xy_(*V*_g_)| in pristine TLG/WSe_2_ as a function of *B* at *T* = 2 K. Only integer Hall plateaus appear, implying the absence of FQHE. f) The top (bottom) panel shows a plot of ρ_D_ versus *B* for TLG/V‐WSe_2_ (TLG/WSe_2_). The non‐monotonic decrease of ρ_D_ with *B* in TLG/V‐WSe_2_ indicates spin‐splitting of the N = 0 LL, while the monotonic increase of ρ_D_ in pristine TLG/WSe_2_ suggests valley‐split N = 0 LL. The dashed line is a visual guide to the eye.

**Figure** **1**a illustrates the device schematic of the ABA‐TLG/V‐WSe_2_ heterostructure. The ABA stacking of TLG was confirmed through Hall measurements, which revealed both monolayer and bilayer filling factors ν  = 2, 6, 8, 10 (Figure S1, Supporting Information).^[^ ^33^ ^]^ The device structure consists of a bottom h‐BN substrate, on which we sequentially transfer the TLG, V‐WSe_2_, followed by the top h‐BN layer. A Graphite flake (∼5 nm) was used as the bottom gate electrode. Holes transferred from V‐WSe_2_ generate hole accumulation in the TLG channel, which is apparent from the upshift of the Dirac point to *V*_D_ = +4.2 V (Figure 1b). In contrast, the pristine TLG/WSe_2_ control sample exhibits only a marginal upshift of *V*_D_ = +1.1 V (inset of Figure 1b). We confirm that conduction takes place only through the TLG layer in the TLG/V‐WSe_2_ heterostructure from transport measurement in monolayer V‐WSe_2_ (Figure S2, Supporting Information).

Figure 1c displays the magnetotransport measurements in the hole regime of the TLG/V‐WSe_2_ heterostructure, performed at a gate bias *V*_g_− *V*_D_ = − 1.7 V and *T* = 2 K. The Hall resistance *R*_xy_ displays a quantized plateau at $-\frac{1}{5/2}\frac{h}{e^{2}}$ in a negative perpendicular magnetic field *B* (Figure 1c), confirming the presence of even‐denominator 5/2 FQHE in the hole regime. Another notable feature is the appearance of a plateau at $R_{\mathrm{xy}}=-\frac{1}{3}\frac{h}{e^{2}}$, situated between the quantized plateaus at $-\frac{1}{2}\frac{h}{e^{2}}$ and $-\frac{1}{5/2\;}\frac{h}{e^{2}}$ (Figure 1c). This reentrant behavior of *R*_xy_ demonstrates RIQHE in TLG/V‐WSe_2_, in addition to 5/2 FQHE. Previously, RIQHE in graphene was observed in *N*  =  2 LL of monolayer graphene.^[^ ^11^ ^]^ We note that FQHE and RIQHE are absent at positive magnetic fields in the hole regime (Figure 1c). Low field (*B* < 3 T) longitudinal resistivity ρ_xx_ exhibit quantum oscillations, which could indicate presence of induced spin‐orbit coupling (SOC) in the TLG.^[^ ^34^ ^]^ A fast Fourier transform of the oscillations (Figure S3, Supporting Information) reveals a single peak, which usually signals the absence of strong SOC in the TLG.

Further confirmation of the ν  = 5/2 fractional state is provided by Hall measurements shown in Figure 1d, obtained by varying the gate bias *V*_g_ at *B*  =   − 8 T. The Hall conductivity |σ_xy_| exhibits a distinct plateau at $\frac{5\;}{2\;}\frac{e^{2}}{h}$ on the hole side, accompanied by a corresponding minimum in the longitudinal resistivity ρ_xx_. The observation of the 5/2 fractional state in another TLG/V‐WSe_2_ sample (device 2) is also shown in Figure S4 (Supporting Information). Additionally, |σ_xy_| displays unusual behavior: i) plateaus appear at different filling factors on the electron and hole side (±*V*_g_), and ii) the plateaus appear in steps of $\frac{3\;e^{2}}{h}$ on the hole side and $\frac{4\;e^{2}}{h}$ on the electron side. This asymmetry in the Hall plateaus at ±*V*_g_ suggests electron–hole asymmetry in the TLG/V‐WSe_2_ sample.

Meanwhile, FQHE is absent in a pristine TLG/WSe_2_ heterostructure (Figure 1e), where only IQHE plateaus are observed. This indicates that V‐doping is crucial in inducing strong correlations necessary to host FQHE and RIQHE in TLG/V‐WSe_2_. On the electron side (Figure 1e), twofold degeneracy of the Hall plateaus is observed with filling sequence of ν  = 2, 4 and 6. The twofold degeneracy is attributed to lifting of the spin/valley degeneracy by the induced SOC (from WSe_2_) in TLG/WSe_2_.^[^ ^35^, ^36^ ^]^ While in the hole regime, the Hall plateaus still appear in steps of $\frac{3e^{2}}{h}$ (Figure 1e), similar to that observed in TLG/V‐WSe_2_. Typically, IQHE plateaus in graphene appear in steps of $\frac{4e^{2}}{h}$ (for *N* > 1 LL) owing to its spin‐valley degeneracy.^[^ ^37^ ^]^ Therefore, the observation of quantized Hall plateaus in steps of $\frac{3e^{2}}{h}$ in both TLG/V‐WSe_2_ and TLG/WSe_2_ suggests the presence of threefold degeneracy in the TLG. Such threefold degeneracy is common in ABA‐TLG and arises from the formation of additional Dirac cones around *K*± points, termed “Dirac gullies”.^[^ ^38^, ^39^ ^]^ Previous studies have shown that Dirac gullies in ABA‐TLG result in quantized Hall plateaus at ν  =   ±3,   ±6,   ±9 and ±12.^[^ ^38^, ^39^ ^]^ We note that the threefold degeneracy in the TLG persists even in the presence of proximate WSe_2_ or V‐WSe_2_.

The band structure of ABA‐TLG consists of a combination of linear monolayer graphene (MLG) and parabolic bilayer graphene (BLG) subbands.^[^ ^40^, ^41^ ^]^ A perpendicular displacement field *D* induces asymmetry in the top and bottom layers of ABA‐TLG, leading to hybridization of these two subbands. Furthermore, the skewed interlayer hopping parameter γ_3_, leads to trigonal warping of the band structure.^[^ ^39^, ^42^ ^]^ At sufficiently high *D*, strong trigonal warping generates three additional Dirac gullies, giving rise to threefold degeneracy.^[^ ^38^, ^39^, ^43^, ^44^ ^]^ In conventional h‐BN encapsulated graphene heterostructures, dual gate (top and bottom) are typically employed to apply *D* (= ε_T_ *V*_T_/*d*_T_ − ε_B_ *V*_B_/*d*_B_) to the graphene layer,^[^ ^38^, ^39^ ^]^ where ε_T_(ε_B_), *V*_T_(*V*_B_) and *d*_T_(*d*_B_) are the dielectric constant, gate voltage and thickness of the dielectric for the top (bottom) layer, respectively. However, in our samples, only a bottom gate is present.

We propose that the bottom gate, in conjunction with hole transfer from V‐WSe_2_ (or WSe_2_) overlayers (to the TLG channel), induces the asymmetry between the top and bottom layers of the TLG, mimicking the effect of a dual gate. Given that the screening length in graphene (∼4 Å) is comparable to the interlayer spacing (∼3.4 Å),^[^ ^45^ ^]^ it is reasonable to assume that the transferred holes from V‐WSe_2_ or WSe_2_ (bottom gate) predominantly reside on the top (bottom) layer of the TLG. Such hole accumulations in TLG render the top and bottom layers asymmetric. We can model the hole transfer from V‐WSe_2_ as being produced by a constant virtual positive voltage (+*V*_T_) directly connected to the top TLG layer, leading to accumulation of holes in the TLG. The effect of +*V*_T_ and the bottom *V*_g_ generates an effective *D* in TLG/V‐WSe_2_. The magnitude of *D* decreases as *V*_g_ is swept toward positive voltages (electron side), as seen from the equation above. Consequently, the observed threefold degeneracy (which is a signature of Dirac gullies) is attributed to this effective *D*, which acts on the TLG.

The Dirac gullies lead to unconventional integer Hall plateaus in the electron regime of TLG/V‐WSe_2_ (Figure 1d). |σ_xy_| plateaus appear with fourfold degeneracy (ν  = 7, 11, and 15), in contrast to the threefold degeneracy observed in the hole regime. However, the observed sequence of filling factors (in the electron regime) is unconventional, as the typically observed filling factors for fourfold degenerate TLG are ν  = 6, 10, 14.^[^ ^46^ ^]^ Interestingly, at high −*B* (> −10 T), TLG/V‐WSe_2_ eventually exhibits a conventional fourfold degenerate filling sequence (i.e., ν  = 6, 10, 14) in the electron regime (Figure S5, Supporting Information). The unconventional sequence of filling factors can be explained by considering contributions from the LLs of the Dirac gullies as well as the central Dirac cone to the IQHE. Thus, the total filling factor is the sum of the filling factors of the threefold degenerate Dirac gullies and the fourfold degenerate central Dirac cone.^[^ ^47^ ^]^ As −*B* and +*V*_g_ increase, the Dirac gullies gradually disappear, and the effect of the central Dirac cone at *K*± becomes prominent (see Note S1 and Figure S6, Supporting Information). This leads to the unconventional filling factors on the electron side (see Note S1, Supporting Information for details). The disappearance of the Dirac gullies on the electron side (+*V*_g_) at high −*B* results from decreasing *D* as the bottom gate is swept toward positive *V*_g_.

It is well established that the formation of Dirac gullies in TLG is favored under a combination of high electric field and low *B*,^[^ ^43^ ^]^ while at high *B*, inter‐gully tunneling suppresses the Dirac gullies.^[^ ^39^ ^]^ For instance, in dual‐gated TLG, Dirac gullies appear at low *B*  = 1.25 T.^[^ ^38^, ^39^ ^]^ However, in our samples, robust Dirac gullies are observed on the hole side at significantly higher fields, up to −14 T in TLG/V‐WSe_2_ (Figure S5, Supporting Information) and − 9 T in TLG/WSe_2_ (Figure 1e), whereas they disappear at high fields on the electron side (Figure S5, Supporting Information). This observation suggests that the presence of V‐WSe_2_ and WSe_2_ modifies the band structure of the TLG, enabling the sustenance of Dirac gullies at higher fields in the hole regime.

The sequence of Hall plateaus |σ_xy_| on the hole side for TLG/V‐WSe_2_ is 5/2, 6, 9, 12, 15, and 18 (Figure 1d), whereas it is 2, 5, 8, 11, and 14 for pristine TLG/WSe_2_ (Figure 1e). The different LL filling sequences in the two samples likely arise from differences in the splitting mechanism of the *N*  =  0 LL. This is reflected in the behavior of the resistance at the Dirac point ρ_D_ as a function of *B*. TLG/V‐WSe_2_​ exhibits a non‐monotonic decrease of ρ_D_ with increasing *B* (upper panel of Figure 1f), which is in stark contrast to the monotonic increase of ρ_D_ in the pristine TLG/WSe_2_ (bottom panel of Figure 1f). This non‐monotonic decrease of ρ_D_ in TLG/V‐WSe_2_ is attributed to spin‐splitting of the *N* = 0 LL, whereas the monotonic increase of ρ_D_ in the pristine TLG/WSe_2_ is consistent with valley‐splitting.^[^ ^48^ ^]^ The emergence of unconventional FQHE and RIQHE in TLG/V‐WSe_2_, driven by ferromagnetic proximity from the V‐WSe_2_ layer, has not been observed previously.

**Figure 2:**
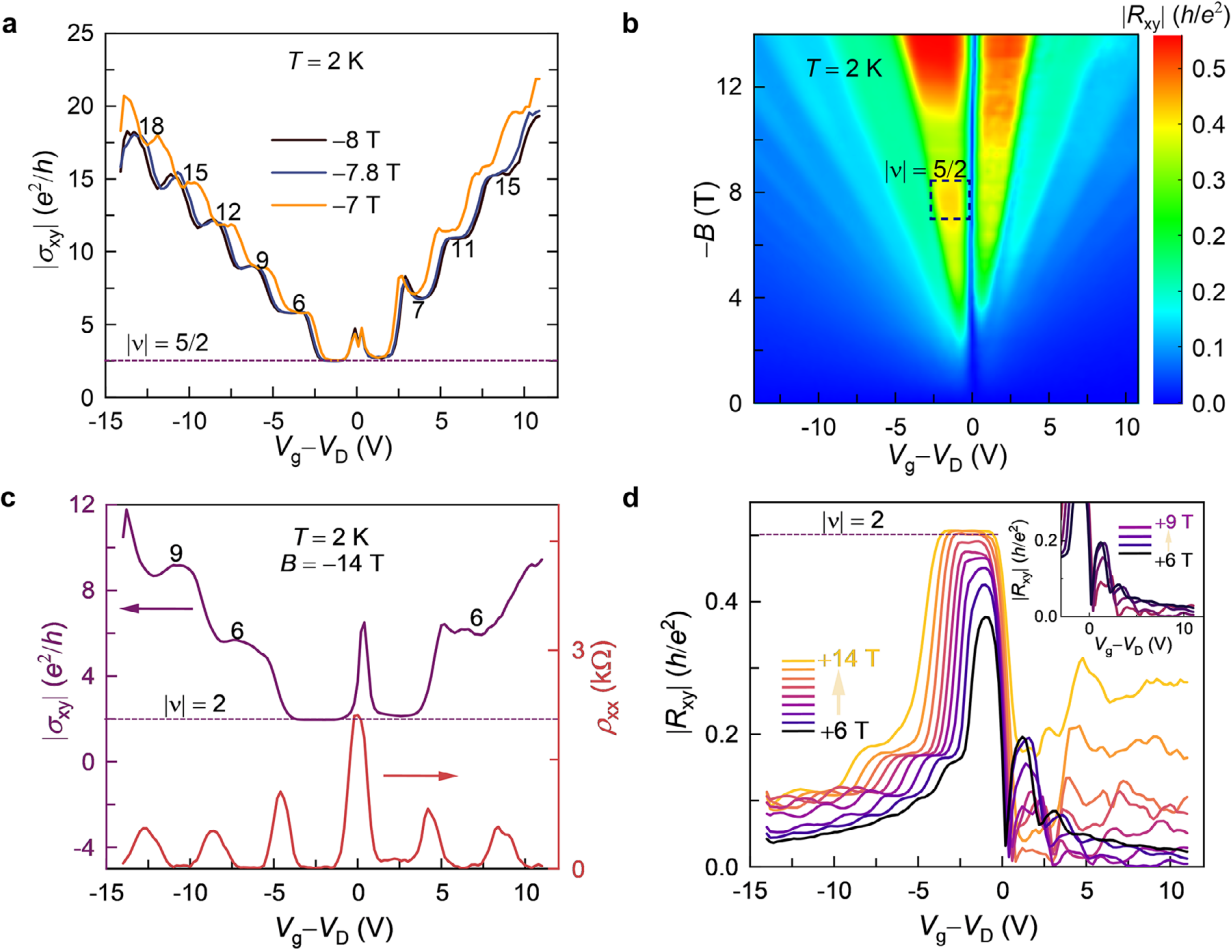
Absence of FQHE at +*B* and higher − *B*. a) |σ_xy_(*V*_g_)| in the field range of − 7 T to − 8 T at 2 K. The 5/2 fractional state on the hole side only persists in this field range. b) Map of |*R*_xy_(*V*_g_, *B*)| measured at *T* = 2 K. The 5/2 fractional state on the hole side only survives in the field range of −7 T to −8 T, corresponding to the region enclosed by the dashed box. c) |σ_xy_| and ρ_xx_ as a function of *V*_g_ at *B* = − 14 T. FQHE is absent at high −*B* and only integer plateaus are observed. d) |*R*_xy_(*V*_g_)| in positive magnetic fields at *T* = 2 K. The absence of FQHE is evident from the appearance of only integer Hall plateaus on the hole side. The plateaus appear with fourfold degeneracy, indicating the absence of Dirac gullies. Meanwhile, Hall plateaus are absent on the electron side; instead, *R*_xy_ oscillates with decreasing amplitude as *V*_g_ is increased. The inset depicts *R*_xy_ oscillations on the electron side in the field range of +6 T to +9 T.

**Figure** **2**a,b displays the results of Hall measurements in negative magnetic fields. On the hole side, the |ν|  = 5/2 state persists only in a narrow field range of −7 T ≤ *B* ≤ − 8 T. In the same field range, TLG/V‐WSe_2_ exhibits crossing of LLs in the Landau fan diagram (Figure S7a, Supporting Information). Such crossing of LLs is known to enhance electron interactions, leading to phenomena such as quantum Hall ferromagnetism^[^ ^41^ ^]^ and FQHE^[^ ^49^ ^]^ in TLG. Thus, the emergence of the 5/2 state is consistent with the presence of strong electron interaction in this field range. At higher fields, the 5/2 fractional state on the hole side is replaced by an integer quantum Hall state |ν|  =  2 (Figure 2c).

Figure 2d shows the Hall resistance |*R*_xy_| measured in positive magnetic fields. Unlike at negative magnetic fields, two key differences are observed in |*R*_xy_|: i) the absence of FQHE (see also Figure S1, Supporting Information) and ii) the lack of Dirac gullies with threefold degenerate LLs on the hole side. Moreover, no LL crossings are observed in the Landau Fan diagram (Figure S7b, Supporting Information), corroborating the lack of strong electron interactions in positive magnetic fields. These results reveal magnetic asymmetry in the strong correlation effects of TLG/V‐WSe_2_. This suggests that strong correlations are favored only for a specific orientation of magnetization. Given that V‐WSe_2_ exhibits ferromagnetic order (see Note S2 and Figure S8, Supporting Information) at the measurement temperature (2 K),^[^ ^50^, ^51^ ^]^ proximity‐induced ferromagnetism likely plays a crucial role in the observed strong correlations in TLG.

Ferromagnetism in V‐WSe_2_ was confirmed by magnetic circular dichroism (MCD) measurements (see Figure S8, Supporting Information). The magnetic hysteresis loop of the TLG/V‐WSe_2_ sample obtained from MCD measurements exhibits exchange bias (see Note S2 and Figure S8b, Supporting Information). The exchange bias can be explained exclusively by the presence of proximity‐induced magnetic order in the TLG and the consequent exchange coupling between the TLG and ferromagnetic V‐WSe_2_ at the interface^[^ ^52^ ^]^ (see Note S2, Supporting Information). This result is consistent with the observation of spin‐split *N* = 0 LL in TLG/V‐WSe_2_ (top panel of Figure 1f), which indicates the presence of a strong interfacial magnetic exchange field (see Note S3, Supporting Information). Such spin‐split *N* = 0 LL due to the exchange field was previously observed in EuS/graphene heterostructure, where the interfacial magnetic exchange field exceeded 14 T.^[^ ^48^ ^]^ Thus, the MCD and transport measurements in TLG/V‐WSe_2_ establish the presence of proximity‐induced magnetism in TLG. Furthermore, non‐local measurements carried out in TLG/V‐WSe_2_ reveal ferromagnetic (canted antiferromagnetic) alignment of spins in the TLG at −*B* (+*B*) (see Note S3 and Figures S9–S11, Supporting Information).

Additionally, the absence of FQHE in pristine TLG/WSe_2_ highlights the critical role of induced magnetization in the TLG layer for the realization of FQHE and RIQHE in TLG/V‐WSe_2_. Spin‐orbit coupling is essential for long‐range ferromagnetism to survive in V‐WSe_2_.^[^ ^50^ ^]^ It is known that SOC plays an important role in promoting strong correlations in graphene. SOC arising from WSe_2_‐interfaced graphene has been known to enhance strongly correlated phenomena such as superconductivity.^[^ ^53^, ^54^, ^55^ ^]^ Therefore, the observed FQHE and RIQHE in TLG/V‐WSe_2_ could be a result of magnetism and SOC working in tandem, which requires further investigation.

Meanwhile, only IQHE is observed at +*B* on the hole side (Figure 2d). Moreover, these IQHE plateaus exhibit fourfold degeneracy, indicating the absence of Dirac gullies at +*B*. Even more intriguing is the absence of Hall plateaus on the electron side (+*V*_g_) under +*B* (Figure 2d; Figure S1, Supporting Information). At high *B*, *R*_xy_ exhibits oscillations with decreasing amplitude as +*V*_g_ increases. The inset in Figure 2d depicts the *R*_xy_ oscillations on the electron side in the field range of +6 T– +9 T. At even higher *B* (≥ +13 T), *R*_xy_ no longer changes sign around the Dirac point and instead maintains negative values across the entire range of ±*V*_g_ (Figure S1, Supporting Information). In previous studies, quantum oscillations in *R*_xy_ have been attributed to the presence of electron–hole pockets in the Fermi surface.^[^ ^56^ ^]^ Therefore, the observed *R*_xy_ oscillations and the lack of sign change around the Dirac point strongly suggest the presence of hole pockets in the conduction band of TLG/V‐WSe_2_.

**Figure 3:**
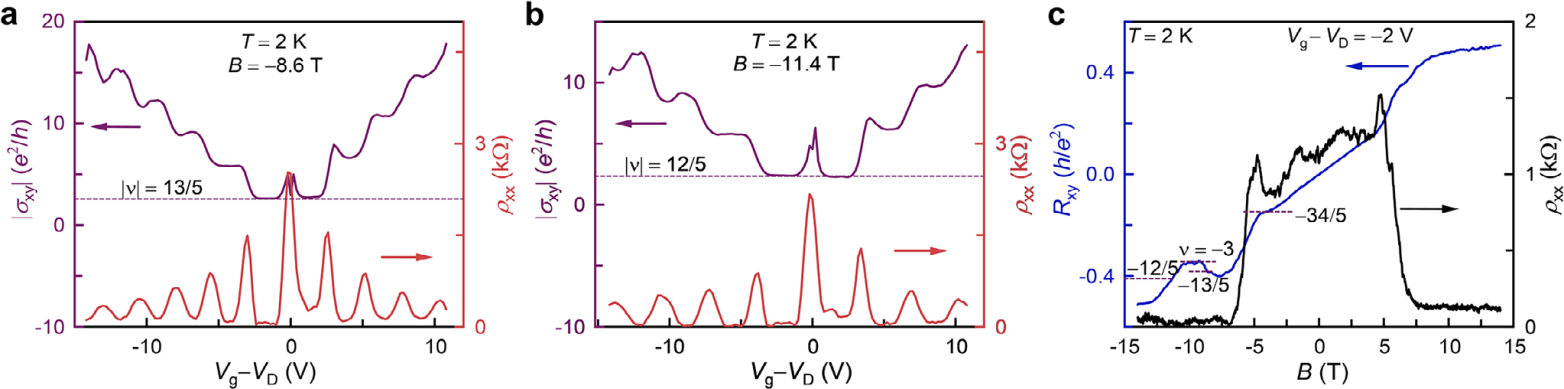
Additional fractional states in TLG/V‐WSe_2_. |σ_xy_| shows distinct plateaus at a) |ν|  = 13/5 and b) |ν|  = 12/5 on the hole side. *V*_g_ scans in a,b) were performed at *B* = −8.6 T and −11.4 T, respectively. c) *R*_xy_ and ρ_xx_ measured as a function of *B* at *V*_g_− *V*_D_ = −2 V and *T* = 2 K. The Hall plateaus are indicated by dashed lines.

In addition to the 5/2 state on the hole side, several other fractional states emerge in the TLG/V‐WSe_2_ sample. **Figure** **3**a,b display |σ_xy_| as a function of *V*_g_ at *B* = −8.6 T and *B* = −11.4 T, respectively. Hall plateaus corresponding to |ν|  = 13/5 and |ν|  =  12/5 fractional states are clearly visible on the hole side. A magnetic field scan on the hole side (Figure 3c) at *V*_g_− *V*_D_ = −2 V reveals the ν  =   −13/5 state as a small plateau in *R*_xy_ accompanied by a corresponding dip in ρ_xx_. Although the plateau in *R*_xy_ for the ν  =   −12/5 is rather obscure in Figure 3c, a clear minimum in ρ_xx_ is observed at *B* = −11.4 T. The minimum in ρ_xx_ in Figure 3c along with the distinct plateau at |ν|  = 12/5 in Figure 3b, both at *B* = −11.4 T, confirm the presence of 12/5 state on the hole side. Additionally, a developing plateau in *R*_xy_ is visible at ν  = −34/5, with a corresponding minimum in ρ_xx_ (Figure 3c).

**Figure 4:**
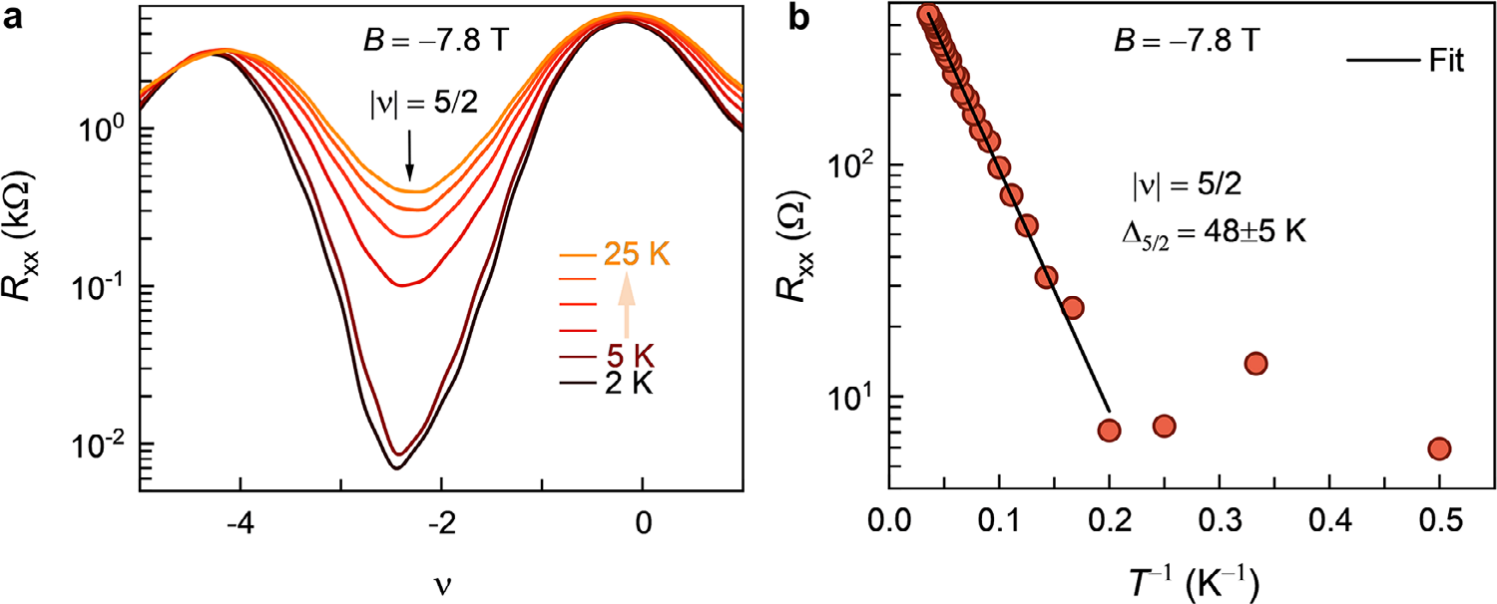
Activation energy gap of the 5/2 fractional state. a) Temperature‐dependent *R*_xx_ measured around |ν|  = 5/2 on the hole side at *B* = − 7.8 T. b) *R*_xx_ minimum plotted as a function of *T* ^−1^ in the temperature range of 2–28 K. The solid black line is the fit using the Arrhenius relation $R_{\mathrm{xx}}∼e^{-\frac{\Delta }{2k_{B}T}}$ (in the region *T* ^−1^ ≤ 0.2 K^−1^). A remarkably large activation energy gap of Δ_5/2_ = 48 ± 5 K is obtained.

For even‐denominator fractional states, the activation energy gap is of paramount importance. The activation energy gap is determined using the Arrhenius relation. $R_{\mathrm{xx}}∼e^{-\Delta /2k_{B}T}$, where *k*_B_ is the Boltzmann constant, and *T* is the temperature.^[^ ^30^, ^57^ ^]^ **Figure** **4**a displays the temperature dependence of *R*_xx_ for the |ν|  =  5/2 state at *B* =   −7.8 T on the hole side. A plot of *R*_xx_ minimum (in the temperature range of 2–28 K) versus *T* ^−1^ is shown in Figure 4b. A linear fit (in the region *T* ^−1^ ≤ 0.2 K^−1^) using the Arrhenius relation (Figure 4b) yields Δ_5/2_ = 48 ± 5 K. To the best of our knowledge, this is the highest reported value for Δ_5/2_, significantly surpassing the previously reported value of ∼1 K in semiconductors 2DEG^[^ ^32^ ^]^ and BLG.^[^ ^16^ ^]^ The remarkably large Δ_5/2_ ensures a very stable 5/2 fractional state. This, in turn, will enable experiments to probe its non‐Abelian nature via quasiparticle exchange (known as braiding) with greater accuracy. Furthermore, the large Δ_5/2_ opens exciting opportunities for fault‐tolerant topological quantum computation. In topological quantum computation utilizing non‐Abelian anyons, the logic gate operations are performed by quasiparticle exchange. The primary source of computational errors arises from thermally generated quasiparticles, which may inadvertently braid with quasiparticles of the computational system, leading to spurious qubits (analogous to bit‐flip errors in classical computation).^[^ ^58^ ^]^ At low temperatures, the density of thermally generated quasiparticles follows $n_{qp}∼\exp\left(-\Delta /2k_{B}T\right)$.^[^ ^30^ ^]^ Consequently, higher values of Δ exponentially suppress error rates. The observed large Δ_5/2_ in this study will enable topological quantum computation with exponentially reduced error rates and higher operational temperatures.

## 3 Conclusion

In summary, we have observed both odd‐ and even‐denominator FQHE along with RIQHE in a TLG/V‐WSe_2_ heterostructure at *T* = 2 K. These strongly correlated phases are attributed to the magnetic proximity effect induced in the TLG by the V‐WSe_2_ overlayer. Notably, we report a remarkably high activation energy gap (Δ_5/2_ = 48 ± 5 K) for the even denominator fractional state, which, to the best of our knowledge, is the highest value reported to date. This remarkably large Δ_5/2_ provides an opportunity to probe the non‐Abelian nature of the 5/2 state in TLG, which has implications for fault‐tolerant topological quantum computation. Furthermore, we observe changes in the Fermi surface topology of the TLG, characterized by the emergence of additional Dirac cones, referred to as Dirac gullies. These Dirac gullies manifest as threefold degenerate Landau levels in magnetotransport measurements and arise due to charge transfer from the V‐WSe_2_ overlayer. Together, these findings not only deepen our understanding of strongly correlated electronic phases in magnetic proximitized graphene‐based heterostructures but also pave the way for studying the non‐Abelian physics in TLG.

## 4 Experimental Section

### Device Fabrication

Graphene, 0.1% V‐doped WSe_2_ (V‐WSe_2_), WSe_2_, and h‐BN flakes were exfoliated on a 300 nm SiO_2_/Si substrate. Trilayer flakes of graphene and monolayer flakes of V‐WSe_2_ and WSe_2_ were identified with the help of an optical microscope. Single crystals of V‐WSe_2_ were synthesized by the chemical vapor transport method. Details of the growth process have been described previously.^[^ ^59^, ^60^ ^]^ The heterostructures of h‐BN encapsulated TLG/V‐WSe_2_ and TLG/WSe_2_ were fabricated using the van der Waals pickup method using polypropylene carbonate (PPC).^[^ ^61^ ^]^ A Graphite flake (∼5 nm) was used as an electrode for the bottom gate. An attempt was made to align the straight edges of graphene and the bottom h‐BN (0°) of both heterostructures. However, secondary Dirac peaks (usually accompanying such 0° graphene‐h‐BN alignment^[^ ^62^ ^]^) were absent in the ρ_xx_ versus V_g_ plot (Figure 1b). This implies that the graphene‐h‐BN edges were inadvertently misaligned (>2°)^[^ ^63^ ^]^ during the fabrication process. Electron beam lithography (EBL) was used to define the hall bar geometry. Oxygen and SF_6_ plasma were used to etch the heterostructure. Electrical contacts were established with Cr/Au deposited using thermal evaporation.

### Magnetotransport Measurement

The magnetotransport measurements were performed in a Quantum Design physical property measurement system with a 14 T superconducting magnet at 2 K. The magnetic field was applied perpendicular to the sample plane for all magnetotransport measurements. Low‐frequency (17.77 Hz) lock‐in (SR830) technique was used to measure the longitudinal (*V*_xx_) and the transverse (*V*_xy_) voltage. The Keithley 6221 current source was used to supply an AC current of 20 nA (at 17.77 Hz) to the sample.

## Conflict of Interest

The authors declare no conflict of interest.

## Author Contributions

P.G. conceived the idea and led the project with the guidance of Y.H.L. P.G. designed the experiments with input from Y.H.L. P.G. carried out all device fabrication with the help of A.M. P.G. performed all the measurements. M.B. synthesized the V‐WSe_2_ single crystals. J.K. performed the MCD measurement. J.L. supervised and advised on the MCD measurement. K.W. and T.T. provided the h‐BN single crystals. P.G. analyzed and interpreted the experimental data. P.G. and Y.H.L. wrote the manuscript. P.G., A.M., M.B., and Y.H.L. discussed the results and commented on the manuscript.

## Supporting information



Supporting Information

## Data Availability

The data that support the findings of this study are available from the corresponding author upon reasonable request.
