# Supplementary material for: Observation of ν = 5/2 Fractional Quantum Hall Effect in Trilayer Graphene Proximitized by V‐Doped WSe2
Source: Adv Mater. 2025 Sep 13;38(1):e14268. doi: 10.1002/adma.202514268 (PMC12759253; doi:10.1002/adma.202514268)
Supplement: Supplementary file 1 — Supporting Information [file ADMA-38-e14268-s001.docx]

**Supporting Information**

**Observation of** $\boldsymbol{\nu}$ **= 5/2 Fractional Quantum Hall Effect in Trilayer Graphene Proximitized by V-Doped WSe_2_**

*Pramod Ghising*, Ashok Mondal, Mallesh Baithi, Jongchan Kim, Jieun Lee, Kenji Watanabe, Takashi Taniguchi, and Young Hee Lee**

P. Ghising, A. Mondal, M. Baithi and Y. H. Lee

Center for Integrated Nanostructure Physics, Sungkyunkwan University

Suwon 16419, Republic of Korea

Email: [pghising@skku.edu](mailto:pghising@skku.edu); [leeyoung@skku.edu](mailto:leeyoung@skku.edu)

A. Mondal, M. Baithi and Y. H. Lee

Center for Low-Dimensional Quantum Materials, Hubei University of Technology

Wuhan 430062, China

Y. H. Lee

Center for Two-dimensional Quantum Heterostructures, Institute for Basic Science (IBS) Sungkyunkwan University,

Suwon 16419, Republic of Korea

A. Mondal, M. Baithi and Y.H. Lee

Department of Energy Science, Sungkyunkwan University

Suwon 16419, Republic of Korea

J. Kim and J. Lee

Department of Physics and Astronomy, and Institute of Applied Physics

Seoul National university

Seoul 08826, Republic of Korea

K. Watanabe

Research Center for Electronic and Optical Materials, National Institute for Materials Science

1-1 Namiki, Tsukuba 305-0044, Japan

T. Taniguchi

Research Center for Materials Nanoarchitectonics , National Institute for Materials Science

1-1 Namiki, Tsukuba 305-0044, Japan

This Supporting Information contains

1. Note S1
2. Note S2
3. Note S3
4. Figures S1-S11

**Note S1: Unconventional filling factors in TLG**

The unconventional filling factor of the TLG on the electron side (Figure 1d) can be explained if we consider the Dirac gullies to gradually disappear with increasing $-$*B* and +*V*_g_. In such a scenario, the three additional Dirac gullies shrink on increasing $-$*B* and +*V*_g_, and the effect of the central Dirac cone at *K*$\pm$ becomes prominent. The central Dirac cone (CD) is fourfold degenerate, while the additional Dirac gullies (AD1, AD2 and AD3) are threefold degenerate. The LLs from the central Dirac cone as well as the Dirac gullies contribute to the IQHE. Thus, the total filling factor $\nu_{T}$ is the sum of the filling factors^[1]^ of the threefold degenerate Dirac gullies ($\nu_{\mathrm{AD}})$ and the fourfold degenerate central Dirac cone ($\nu_{\mathrm{CD}})$, i.e., $\nu_{T}=\nu_{\mathrm{AD}}+\nu_{\mathrm{CD}}$*.*

With an increase in $-$*B* and +*V*_g_, the three Dirac gullies gradually shrink (Figure S6). Therefore, as +*V*_g_ is increased, only the lowest LL is populated in the Dirac gullies (as they gradually shrink), whereas higher LLs in the central Dirac cone are populated (Figure S6). Therefore, $\nu_{\mathrm{AD}}=$ 3 (since only the lowest threefold degenerate LL is populated at all +*V*_g_), whereas for the fourfold degenerate central Dirac cone, $\nu_{\mathrm{CD}}=$ +4, +8, +12, as higher LLs are populated with increasing +*V*_g_. Thus, the sequence of filling factors on the electron side comes out to be $\nu_{T}=\nu_{\mathrm{AD}}+\nu_{\mathrm{CD}}$ i.e., 3+4 $=$ 7, 3+8 $=$ 11, 3+12 $=$ 15. At high *B*, the Dirac gullies disappear completely and only the LLs from the fourfold degenerate central Dirac cones contribute to the IQHE. Consequently, at high *B*, $|\sigma$_xy_$|$ filling factors appear like that in a conventional fourfold degenerate TLG i.e., 6, 10 and 14 (Figure S5), indicating the presence of only the central Dirac cone. This demonstrates that the trigonal warping-induced Dirac gullies disappear (on the electron side) at high $-$*B* and +*V*_g_.

**Note S2: Magnetic circular dichroism (MCD) measurements in V-WSe_2_ and TLG/V-WSe_2_**

Magnetic circular dichroism (MCD) measurements were performed to probe magnetism in hBN-encapsulated V-WSe_2_ (hBN/V-WSe_2_/hBN) and the TLG/V-WSe_2_ samples, with magnetic fields applied perpendicular to the sample plane. MCD measurements were performed using a solid-state laser ($\lambda=$705-720 nm, P $=$ 50 $\mu$W) at 2 K. A photo-elastic modulator was used to modulate the laser between left and right circular polarization. The MCD signal was detected as the difference in absorbance between the left and right circular polarization.

The MCD hysteresis loop of the hBN-encapsulated V-WSe_2_ sample (at 2 K) shown in Figure S8a confirms ferromagnetic order in V-WSe_2_. The MCD hysteresis loop of the TLG/V-WSe_2_ sample (after subtraction of a linear diamagnetic background) at 2 K is shown in Figure S8b. The TLG/V-WSe_2_ sample exhibits two interesting features in the hysteresis loops: i) loop inversion and ii) horizontal loop shift. Both these features are signatures of exchange bias (EB) phenomena, which originate from interfacial exchange coupling of spins^[2,3,4]^. EB is usually observed in magnetic interfaces [ferromagnet (FM)/antiferromagnet (AFM) and FM/FM type], where the exchange coupling gives rise to unidirectional anisotropy in the magnetic system. However, EB was also observed in an FM (LaMnO_3_)/non-magnetic (LaNiO_3_) heterostructure, which was attributed to the exchange coupling between the proximity induced magnetic moments in LaNiO_3_ and the FM moments in LaMnO_3_^[5]^. Therefore, EB in TLG/V-WSe_2_ indicates the presence of proximity-induced magnetism in the TLG which is strongly exchange-coupled to the ferromagnetic V-WSe_2_. The observed EB is consistent with the presence of strong interfacial magnetic exchange field (MEF) which leads to the spin-split *N* $=$ 0 LL in TLG/V-WSe_2_ seen in Figure 1f. Additionally, the MCD hysteresis loop disappears at 200 K (shown in Figure S8c), implying a magnetic phase transition in the TLG/V-WSe_2_ at higher temperatures.

Furthermore, the MCD hysteresis loop of TLG/V-WSe_2_ exhibits an unusual feature at positive magnetic fields (Figure S8b). Above *B* $=$ +2 T, the MCD signal increases monotonically after a saturation like behavior below +2 T. Similar behavior of the magnetic hysteresis loop was previously reported in zigzag edged graphene samples and was ascribed to the presence of different competing magnetic orders i.e., ferromagnetism, antiferromagnetism and diamagnetism^[6]^. In the TLG/V-WSe_2_ sample, canted antiferromagnetic spin phase is observed in the TLG at higher positive magnetic fields [see Supporting Information Note S3 and Figure S11]. Therefore, the monotonic increase in the MCD signal above +2 T likely arises from the onset of canted antiferromagnetic spin phase in the TLG which contributes to the overall magnetization of TLG/V-WSe_2_.

**Note S3: Evidence for ferromagnetic (canted antiferromagnetic) spin phase at** $\boldsymbol{-}$***B*(*+B*) in TLG/V-WSe_2_**

In the TLG/V-WSe_2_ sample, the variation of the resistivity at the Dirac point $\rho_{D}$ with $-$*B* (top panel of Figure 1f in the manuscript) exhibits a non-monotonic decrease, suggesting a spin-polarized $\nu=$ 0 state similar to that observed in EuS/graphene^[7]^. The appearance of spin-polarized state requires enormous Zeeman exchange energy to overcome the coulomb interaction energy^[8,9]^. In EuS/graphene, magnetic proximity form EuS generates very high interfacial magnetic exchange field (MEF), exceeding 14 T^[7]^. Therefore, such spin-polarized state in TLG/V-WSe_2_ can only be explained by the presence of very high interfacial MEF to generate the required exchange splitting. Moreover, such spin-polarization is absent in pristine TLG/WSe_2_ (which instead shows valley-polarization, bottom panel of Figure 1f). Therefore, the high MEF must originate from magnetic V-WSe_2_.

At very high external magnetic fields, the spin-polarized $\nu=$0 state exhibits metallic counter-propagating quantum spin Hall (QSH) like edge states^[9]^. The QSH states have been observed in both ferromagnetic (FM) and canted antiferromagnetic (CAF) spin phase of graphene. The FM phase exhibits a gapless edge state, while the CAF phase features a small gap at the edge^[9]^ (shown in Figure S9).

In negative magnetic fields, $\rho_{D}$($-$*B >* $-$5 T) drops below $\rho_{D}$(*B* $=$ 0) as seen in the upper panel of Figure 1f, indicating the onset of metallic $\nu=$ 0 state. Since at $-$*B* > $-$5 T, TLG/V-WSe_2_ is in the quantum Hall regime, edge states are responsible for conduction, which suggests counter-propagating $\nu=$ 0 edge states^[7]^ in this field range. Meanwhile, the behavior of $\rho_{D}$ is markedly different under positive magnetic fields (see Figure S10). $\rho_{D}$ still exhibits a non-monotonic decrease, signifying $\nu=$ 0 state is still spin-polarized. Notably, $\rho_{D}$(+*B* $\neq0$) is larger than $\rho_{D}$(*B*$=$ 0) for all +*B* except *B* $=$ +14 T, where it drops slightly below the value at *B* $=$ 0 (Figure S10). Note the metallic $\nu=$ 0 state [where $\rho_{D}$(*B* $\neq0$) $<$ $\rho_{D}$(*B* $=$ 0)] is absent under +*B*, which was readily observed at low fields in the case of $-$*B*. This contrasting behavior of $\rho_{D}$ in $\pm$*B* suggests different nature of the edge states at $\pm$*B*. While the low field metallic $\nu=$ 0 state under $-$*B* indicates a gapless edge state; $\rho_{D}$(+*B*$\neq$ 0) $> \rho_{D}$(*B*$=$ 0) suggests the edge state exhibits a gap under +*B*. Since $\rho_{D}$(+*B* $\neq$ 0) is not very high compared to $\rho_{D}$(*B*$=$ 0), we can infer that the gap is small. This implies that the TLG exhibits an FM phase (gapless edge) for negative *B* and a CAF phase (gapped edge) at positive *B*.

To probe the counter-propagating edge states, we measure the non-local resistance in the TLG/V-WSe_2_ sample with different perpendicular magnetic fields (Figure S11). The blue dashed curve depicts the longitudinal resistance $\rho_{\mathrm{xx}}$ at *B* $=$ 0. The non-local resistance *R*_NL_ at *B* $=$ 0 is negligibly small. Whereas *R*_NL_ exceeds the value of $\rho_{\mathrm{xx}}$ for +*B* and is comparable to $\rho_{\mathrm{xx}}$ at $-$*B*. Such high values of *R*_NL_ rules out the possibility of the non-local signal originating from ohmic contribution which is given by $R_{\mathrm{ohmic}}\approx\rho_{\mathrm{xx}}e^{-\pi L/W}$ (*L* and *W* are sample length and width, respectively). Notice that the *R*_NL_ peak for +8 T and +4 T appear slightly away from the Dirac point, this is consistent with a small gap opening at the edge and conduction occurs only when the Fermi level moves away from the Dirac point^[9]^. Thus, the non-local measurements support the presence of a gapped CAF phase in the TLG under positive magnetic fields.

Furthermore, even the *R*_NL_ peak at $-$4 T appears away from the Dirac point. This can be explained by a continuous phase change from CAF to a ferromagnetic (FM) phase (such a continuous phase change from CAF to FM with external *B* has been reported previously^[9]^). At low fields ($-$4 T) a CAF phase is expected, because the spin alignment by $-$*B* has just begun (onset of decreasing $\rho_{D}$ with $-$*B* in top panel of Figure 1f). At high fields ($-$8 T), a broad *R*_NL_ peak is observed at the Dirac point (Figure S11a). The broad *R*_NL_ peak at the Dirac point at $-$8 T indicates gap closure at the edges, signaling the presence of a gapless FM phase in the TLG under negative magnetic fields.

**Figure S1 |** ***R*_xy_ measured as a function of *V*_g_ at positive magnetic fields in TLG/V-WSe_2_**. At *B* > +5 T, only integer Hall plateaus are observed (on the hole side), signifying the absence of FQHE in positive magnetic fields. The filling factors of 2, 6, 8 and 10 indicate the presence of both monolayer and bilayer like LLs, which is typical of ABA-trilayer graphene^[10]^. Interestingly, Hall plateaus are absent on the electron side. Instead, *R*_xy_ exhibit oscillations (on the electron side) with decreasing amplitude. At higher *B*, *R*_xy_ does not change sign at the Dirac point and retains negative value for the entire range of $\pm$ *V*_g_. *R*_xy_ oscillations along with its tendency to abstain from a sign change at the Dirac point (at higher *B*) indicates the presence of electron-hole pockets in the Fermi surface.

**Figure S2|** ***I*_DS_-*V*_g_ characteristics of V-WSe_2_ at 300 K**. *I*_DS_-*V*_g_ characteristics of V-WSe_2_ with Cr/Au contact. The sample resistance at *V*_g_$=$ $-$40 V (which gives the highest current level) is ~3$\times$10^8^ $\Omega$ and the sample mobility and carrier density are 3$\times$10^-2^ cm^2^V^-1^s^-1^ and 1.8$\times$10^12^ cm^-2^, respectively. The mobility is typically low due to the high Schottky barrier height. It is observed from the *I*_DS_-*V*_g_ plot that the resistance increases significantly at low gate voltages. In the TLG/V-WSe_2_ heterostructure, V-WSe_2_ is on top of the TLG. Consequently, *V*_g_ applied by the bottom gate is significantly screened by the intermediate metallic TLG layer, reducing its effect on V-WSe_2_. Therefore, we can conclude that V-WSe_2_ exhibits significantly higher resistance in TLG/V-WSe_2_ heterostructure, ensuring TLG as the primary conduction channel.

**Figure S3|** **Low field quantum oscillation in Hall measurement**. a) Low field quantum oscillations of $\rho$_xx_ (observed in Figure 1c) plotted as a function of 1/*B*. Due to the background noise in the data, smooth oscillations are not observed. b) FFT of the quantum oscillations in (a).

**Figure S4|** **Hall measurement in another TLG/V-WSe_2_ sample (device 2)**. $\sigma_{\mathrm{xy}}$ shows a robust plateau at $\nu=$5/2 on the hole side in a magnetic field range of $-$8 T to $-$10 T.

**Figure S5|** $\boldsymbol{|}\boldsymbol{\sigma}_{\mathbf{xy}}$**(*V*_g_)**$\boldsymbol{|}$ **measured at higher negative magnetic fields**. On the electron side (+*V*_g_), the Dirac gullies completely disappear at high $-$*B*, and only conventional trilayer graphene like filling factors ($\nu=$ 6, 10, 14) are observed. Whereas, on the hole side ($-$*V*_g_), the Dirac gullies persist even at high fields, which is apparent from the threefold degeneracy of the Hall plateaus.

**Figure S6|** **Unconventional filling factor in TLG/V-WSe_2_ on the electron side**. Schematic of filling of the three additional Dirac cones (AD1, AD2, AD3) and the central Dirac cone (CD) with increasing $-$*B* and +*V*_g_ (on the electron side) in TLG/V-WSe_2_. The red dashed line represents the different Landau levels (LL). The three additional Dirac gullies shrink with increasing $-$*B* and +*V*_g_ and the effect of the central Dirac cone (with fourfold degeneracy) becomes prominent. +*V*_g_ populates higher LLs in the central Dirac cone, while only the lowest LLs are populated in the additional Dirac gullies (as Dirac gullies gradually shrink). The total filling factor is the sum of the filling factors of the additional Dirac cones (3 due to the lowest LL filling) and the central Dirac cone (i.e., 4, 8, 12). Thus, the observed filling factor on the electron-side in Figure 1d can be explained as 3+4 $=$ 7, 3+8 $=$ 11 and 3+12 $=$ 15.

**Figure S7|** **Landau Fan diagram of TLG/V-WSe_2_.** a) at $-$*B* and b) at +*B*. In negative magnetic fields the fan diagram shows regions with increased value of *R*_xx_ (enclosed areas with white dashed line). This results from crossing of the monolayer and bilayer LLs^[11,12]^. These crossings occur because of asymmetry in the TLG band structure introduced by perpendicular *D*. This leads to trigonal warping and subsequent Dirac gullies. However, in positive magnetic fields no such crossings are evident.

**Figure S8| Ferromagnetism in V-WSe_2_.** Results of the magnetic circular dichroism (MCD) measurements in a) hBN-encapsulated V-WSe_2_ (hBN/V-WSe_2_/hBN) and b) TLG/V-WSe_2_ at 2K. The MCD hysteresis loop confirms ferromagnetic order in V-WSe_2_. The observed exchange bias (EB) in TLG/V-WSe_2_ (horizontal loop shift) confirms the presence of proximity induced magnetism in the TLG, which exchange couples to the ferromagnetic order in V-WSe_2_. The monotonic increase in the MCD signal beyond +2 T in (b) likely arises from the onset of canted antiferromagnetic spin ordering (CAF phase) in the TLG, which contributes to the overall magnetization of TLG/V-WSe_2_. The arrows in (b) depict the field sweep direction. c) MCD measurement in TLG/V-WSe_2_ at 200 K, the hysteresis disappears at higher temperature.


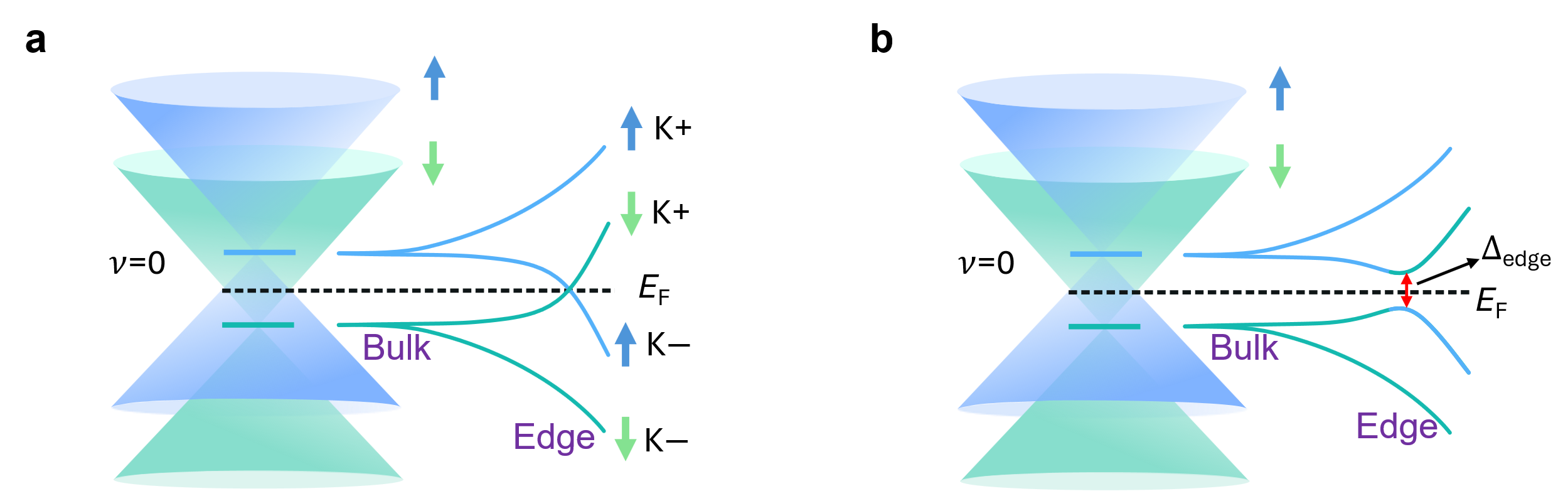


**Figure S9|** **Spin polarized** $\boldsymbol{\nu=0}$**state.** The edge states of the ferromagnetic (FM) phase (a) and the canted antiferromagnetic phase (CAF) phase (b) of the TLG. In the FM phase the edge is gapless, while the CAF phase exhibits a small gap $\Delta$_edge_ at the edge.

**Figure S10|** **Dirac point Resistance** $\boldsymbol{\rho}$**_D_ in TLG/V-WSe_2_ at positive magnetic fields.** $\rho$_D_ exhibits a non-monotonic behavior, indicating a spin-polarized $\nu=$ 0 state under positive *B*. However, $\rho$_D_(*B*$\neq0$) drops below $\rho$_D_(*B*$=$0) only at high *B* $=$ +14 T.

**Figure S11|** **Non-local measurement in TLG/V-WSe_2_ at 2 K.** (a) Non-local resistance *R*_NL_ at different perpendicular magnetic fields. The dashed blue curve depicts the longitudinal resistivity $\rho$_xx_ at *B* $=$ 0 T. (b) Measurement configuration for *R*_NL_. The encircled numbers represent the electrodes depicted in the inset of Figure 1a.

**References**

1. G. Krizman *et al.* *Valley-Polarized Quantum Hall Phase in a Strain-Controlled Dirac System*, Phys. Rev. Lett*.* **132**, 166601 (2024).
2. M. Ziese, I. Vrejoiu and D. Hesse, *Inverted hysteresis and giant exchange bias in La*_0.7_*Sr*_0.3_*MnO_3_/ SrRuO*_3_, Appl. Phys. Lett. **97**, 052504 (2010).
3. J. Nogués and I. K. Schuller, *Exchange bias*, J. Magn. Magn. Mater. **192**, 203 (1999).
4. S. Pradhan, S. Satapathy, M. Nayak, J. R. Mohanty, S. K. Mishra and S. K. Majumder, *Magneto-optical Kerr effect (MOKE) and magnetic force microscopy (MFM) studies on Cr/Ni nanodot arrays deposited using innovative nano-stencil method*, J. Mater. Sci: Mater. Electron **35**, 2094 (2024).
5. M. Gibert, P Zubko, R. Scherwitzl, J. Íñiguez and Jean-Marc Triscone, *Exchange bias in LaNiO*_3_*-LaMnO*_3_ *superlattices*, Nat. Mater. **11**, 195 (2012).
6. L. Chen, L. Guo, Z. Li, H. Zhang, J. Lin, J. Huang, S. Jin and X. Chen, *Towards intrinsic magnetism of graphene sheets with irregular zigzag edges*, Sci. Rep. **3**, 2599 (2013).
7. P. Wei, S. Lee, F. Lemaitre, L. Pinel, D. Cutaia, W. Cha, F. Katmis, Y. Zhu, D. Heiman, J. Hone, J. S. Moodera, C.-T. Chen, *Strong interfacial exchange field in the graphene/EuS heterostructure*, Nat. Mater*.* **15**, 711 (2016).
8. L. Veyrat, C. Deprez, A. Coissard, X. Li, F. Gay, K. Watanabe, T. Taniguchi, Z. Han, B. A. Piot, H. Sellier and B. Sacepe, *Helical quantum Hall phase in graphene on SrTiO*_3_*,* Science **367**, 781 (2020).
9. A. F. Young, J. D. Sanchez-Yamagishi, B. Hunt, S. H. Choi, K. Watanabe, T. Taniguchi, R. C. Ashoori and P. Jarillo-Herrero, *Tunable symmetry breaking and helical edge transport in a graphene quantum spin Hall state*, Nature **505**, 528 (2014).
10. Y. Lee, Jairo Velasco, Jr, D. Tran, F. Zhang, W. Bao, L. Jing, K. Myhro, D. Smirnov and C. N. Lau, *Broken Symmetry Quantum Hall States in Dual-Gated ABA Trilayer Graphene*, Nano Lett. **13**, 1627 (2013).
11. B. Datta, S. Dey, A. Samanta, H. Agarwal, A. Borah, K. Watanabe, T. Taniguchi, R. Sensarma, and M. M. Deshmukh, *Strong Electronic Interaction and Multiple Quantum Hall Ferromagnetic Phases in Trilayer Graphene*, Nat. Commun. **8**, 14518 (2017).
12. T. Taychatanapat, K. Watanabe, T. Taniguchi, and P. Jarillo-Herrero, *Quantum Hall Effect and Landau-Level Crossing of Dirac Fermions in Trilayer Graphene*, Nat. Phys. **7**, 621 (2011).
